# Ensemble technique of intrusion detection for IoT-edge platform

**DOI:** 10.1038/s41598-024-62435-y

**Published:** 2024-05-22

**Authors:** Abdulaziz Aldaej, Imdad Ullah, Tariq Ahamed Ahanger, Mohammed Atiquzzaman

**Affiliations:** 1 College of Computer Engineering and Sciences, Prince Sattam Bin Abdulaziz University, Al-Kharj, 11942 Saudi Arabia; 2 School of Computer Science, Faculty of Engineering, The University of Sydney, Sydney, NSW 2006 Australia; 3 Department of Management Information Systems, CoBA, Prince Sattam Bin Abdulaziz University, Al-Kharj, 11942 Saudi Arabia; 4 School of Computer Science, University of Oklahoma Norman, Norman, 73019-6151 USA

**Keywords:** Edge computing, Internet of things, Ensemble technique, Security, Intrusion detection system, Engineering, Mathematics and computing

## Abstract

Internet of Things (IoT) technology has revolutionized modern industrial sectors. Moreover, IoT technology has been incorporated within several vital domains of applicability. However, security is overlooked due to the limited resources of IoT devices. Intrusion detection methods are crucial for detecting attacks and responding adequately to every IoT attack. Conspicuously, the current study outlines a two-stage procedure for the determination and identification of intrusions. In the first stage, a binary classifier termed an Extra Tree (E-Tree) is used to analyze the flow of IoT data traffic within the network. In the second stage, an Ensemble Technique (ET) comprising of E-Tree, Deep Neural Network (DNN), and Random Forest (RF) examines the invasive events that have been identified. The proposed approach is validated for performance analysis. Specifically, Bot-IoT, CICIDS2018, NSL-KDD, and IoTID20 dataset were used for an in-depth performance assessment. Experimental results showed that the suggested strategy was more effective than existing machine learning methods. Specifically, the proposed technique registered enhanced statistical measures of accuracy, normalized accuracy, recall measure, and stability.

## Introduction

Industrial sectors have been equipped with internet connectivity for provisioning users/customers access to a wealth of information and conveniences. Conspicuously, the notion of Internet of Things (IoT) ecosystems is developing and gaining traction. The number of internet-enabled devices is rising at an enormous pace. Devices in heterogeneous environments vary in size and functionality but have common access to the internet and portability^1,2^. IoT devices are characterized by minimal memory and processing power due to the compact form factors. Therefore, IoT data must be sent to a device with more computing capability to be stored, processed, and analyzed. The transmission to the cloud is impeded by the enormous volume of data produced by these devices and the delay involved. *Edge Computing * is a decentralized platform that processes data at the network’s edge before transmitting it to the cloud for further analysis^3^. As a result, processing and response times for IoT devices may be improved.

**Figure 1:**
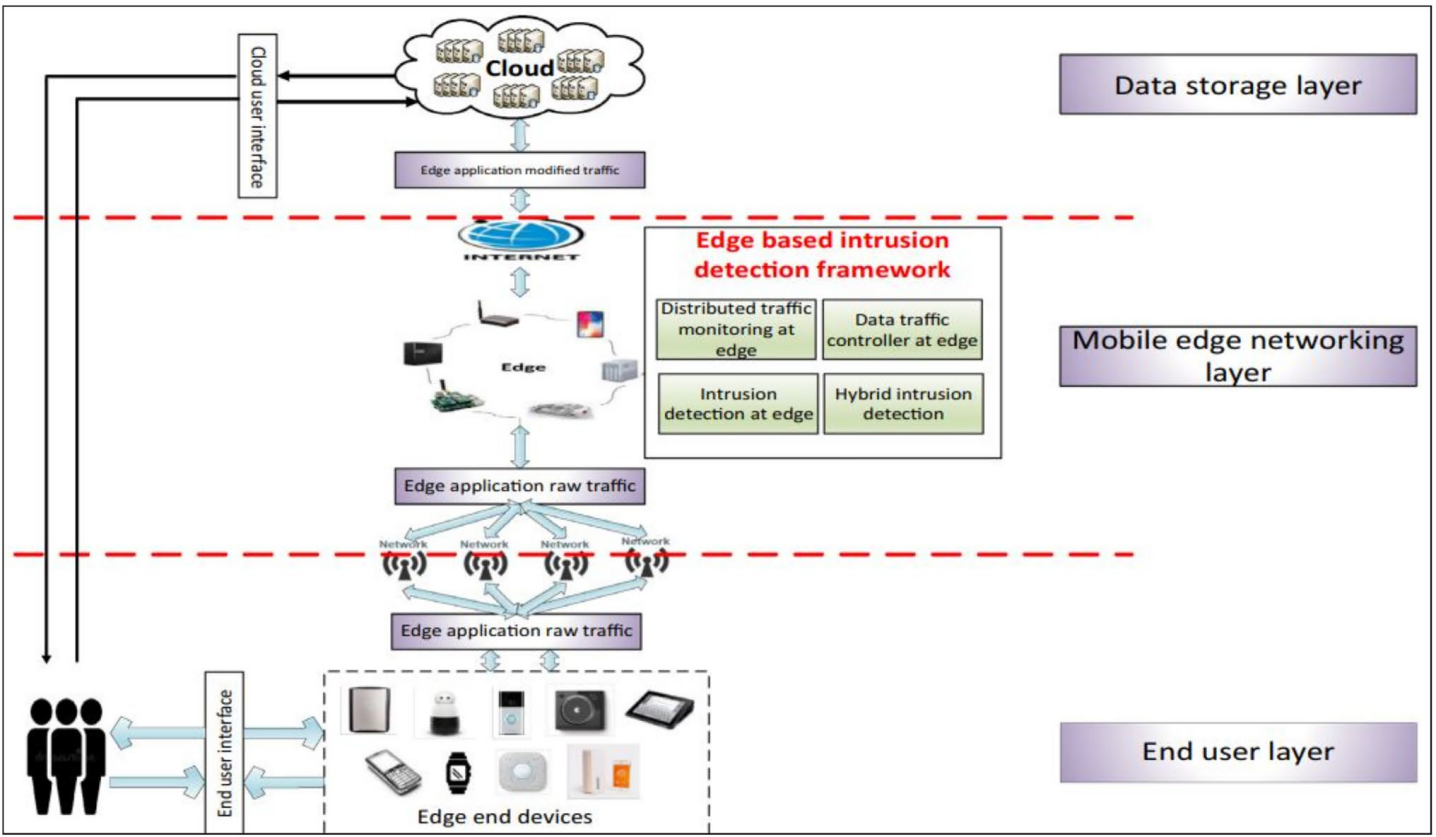
Conceptual overview of edge computing architecture with IDS.

Because of the limited computing power, IoT devices present unique challenges when it comes to implementing security methods^4^. Cases of security breaches are widespread in IoT systems as it is overlooked in the framework design process. Figure 1 shows the conceptual overview of edge computing-based IDS detection^5^. In 2016, hackers used the Mirai virus to launch DDoS attacks on a large number of IoT devices (Source: https://www.theguardian.com/technology/2016/oct/.). Due to the critical nature of today’s computing systems, specialized security measures and intrusion detection strategies are required to detect and prevent malicious activity. There have been some promising developments in the detection approaches that use machine learning. Despite several machine learning procedures, security cannot be guaranteed effectively by technologies that just identify the existence of intrusions (binary detection)^6^. Consequently, it is crucial to determine the kind of attack to devise suitable countermeasures. In addition, the administrator of the network may find the identification helpful when making choices. The administrator has the option of taking measures to close the security hole that was exploited in the attack^7^. Several forms of attacks may be difficult to detect using multi-category determination techniques that try to determine attack class^8^. Existing multi-category detection approaches have reduced success rates than binary techniques. The presented work advances state-of-the-art works significantly. To enhance intrusion detection and identification, the suggested technique makes use of ensemble approaches and a dual analytic architecture. As there are machine learning instances where optimal models are not precise, ensemble learning is employed in the current research proposal. To stabilize models, it is required to mix them utilizing hybrid approaches. The state-of-the-art methods have limitations in determining certain types of attacks. Most were not affected by fluctuations since they were tested using a single database.

### Motivational aspects

The motivational aspects lies in the growing significance of IoT technology in modern industrial sectors and various domains of applicability. While IoT has brought about significant advancements, security concerns have been somewhat overlooked due to the limited resources of IoT devices. The need for effective intrusion detection methods to detect and respond to IoT attacks is crucial. The motivation stems from the necessity to enhance the security of IoT systems and networks, as well as the potential to contribute to the development of more effective and robust intrusion detection methods tailored to the unique challenges posed by IoT environments. Conspicuously, in the current study, an edge computing-based intrusion detection architecture is introduced. The proposed two-stage architecture detection approach seeks to categorize events as either attacks or non-attacks. In the first stage, a binary Extra Tree (E-Tree) classifier is used to analyze the data. Henceforth, it will determine whether or not the traffic flows are invasive. The gates automatically open for traffic that has been deemed safe. The second stage is applied to analyze unwanted traffic. At this stage, a more robust technique that incorporates E-Tree, Random Forest (RF), and Deep Neural Network (DNN) is collaboratively used as an ensemble approach. Moro ever, the traffic is analyzed once again to determine the broad category of incursion. Based on the aforementioned aspects, specific contributions of the current research are as follows;

### State-of-the-art contributions

Based on the aforementioned motivational aspects, the specific research contributions of the paper are depicted ahead;

1. Introducing a novel method for identifying intrusions in a specific IoT environment at the edge by integrating attribute detection, category normalization, training, and ensemble classification.
2. Presenting a two stage intrusion detection mechanism using binary classification approach based on E-Tree.
3. Proposing a wrapper approach that combines E-Tree and Recursive attribute elimination over edge computing for security.
4. Experimentally validating the proposed model and compare the results with state-of-the-art works and machine learning techniques.

Paper organization The fundamental ideas and review of vital literature related to intrusion detection are investigated in “Literature review”. In “Proposed model”, the proposed methodology is described in detail. In “Experimental simulation”, the experimental simulation is performed for performance comparison between the proposed approach and related works. “Conclusion” concludes the paper with possible future directions.

## Literature review

This section discusses methods provided by various studies to protect edge computing and IoT-based frameworks from intrusion detection and identification. The existing research on the subject was analyzed. Elsevier, IEEE and Springer were scoured for relevant articles. In Table 1, we see a comparison of the works that constitute the state-of-the-art research works for determining methods that are capable of multi-category detection and their primary flaws. Using the Support vector Machine and the l1-regularized technique, Ali et al.^10^ suggested a detection methodology for binary and multi-category attacks. To find intruders in an IoT environment, Sasikumar et al.^9^ presented a decentralized method using the DNN technique. The edge layer nodes perform the detecting procedure. Each node is equipped with a DNN detection model, and training takes place across all of the nodes. The NSL-KDD dataset was used for testing purposes exclusively. For binary intrusion detection in edge computing settings, Deng et al.^11^ proposed a hybrid technique using DNN and KNN. For intrusion determination, Duan et al.^17^ assessed at deep networks. A technique using Deep Belief Networks was presented. An intrusion detection method based on Sequential Online ELM is proposed by Gupta et al.^12^. High training speed and strong generalizability are registered by the neural network known as Extreme Learning Machine. OS-ELM is an evolution of ELM networks specifically designed to manage services in the cloud including multi-category detection in the method. Unfortunately, it complicates efforts to identify attacks like probing and privilege escalation. Convolutional Neural Networks were originally developed for imagery classification, however Alam et al.^13^ suggested using CNNs as a multi-category network attack classifier that can be installed in a router. The UNSW-NB15 and the NSL-KDD public data were used to validate the method. However, the IoT context is not addressed in the proposed study. A variant of CNN called Vector Convolutional Deep Learning was suggested by Han et al.^16^ to identify abnormalities in IoT data. As the suggested method employs a vector rather than a matrix, the term “convolutional vector” is used to describe it. For this purpose, the authors utilized the Bot-IoT dataset. Event categorization was suggested by Jasim et al.^18^ using a Deep Recurrent Neural Network technique. To prevent discriminating behavior for classes with important instances, oversampling is used during the training phase of the neural network. The goal is to raise the precision of attacks that have been subjected to fewer training instances. Roy et al.^14^ also experimented with RNN, introducing the Multi-head Attention layer to collect and learn the global representation. Correlated-Set Thresholding and DT are two novel attributes introduced by Ge et al.^15^ that are based on machine learning and have been built for and implemented in Raspberry Pi. The method is tested on the existing Bot-IoT dataset, however there are no findings about the classification of benign. A novel method for attribute selection was presented by Xu et al.^19^. Utilizing the AUC, the new method employs the wrapper technique to precisely filter attributes and pick relevant attributes for the machine learning approach. Using the Bot-IoT dataset and four different ML methods, the suggested method is verified. To boost generalizability and robustness, RFs use a collection of DTs that are integrated into an ensemble. Utilizing a hybrid resource selection mechanism that prioritizes important resources and RF to categorize traffic as normal or anomalous, Majeed et al.^20^ developed an anomaly-based strategy for IoT networks. The IoTID20 anomaly detection dataset was used to measure performance. Unfortunately, the authors registered reduced performance in the context of multi-category detection. Alshamrani et al.^21^ recommended employing the encoding phase of the LSTM to minimize the dimensions of the massive IoT data. To appropriately categorize the network traffic samples, authors utilized deep Bidirectional LSTM to assess the long-term correlated changes in the low-dimensional resource set. Using the BoT-IoT dataset, authors deduced that LAE greatly decreased the amount of memory needed to store massive amounts of network traffic. The method has an 89% success rate in identifying safe traffic, which suggests a rise in the number of false positive results. In the presented article, we have looked at a few different strategies. Several approaches used machine learning techniques to locate and categorize network breaches. IoT technological aspects were ignored by several authors. Several methods had trouble identifying certain forms of attack, while others relied on a single dataset for validation. Henceforth, the promising outcomes of DT-based ensemble techniques need further study.

**Table 1:** Comparative analysis (Y Yes, N No).

References	Method	Multi-category	Limitation	Dataset
^9^	DBN	Y	Low accuracy	KDDCUP 99
^10^	OS-ELM	Y	Low accuracy	NSL-KDD
^11^	SVM+LASSO	Y	Single Data Validation	NSL-KDD
^12^	DNN + KNN	Y	Single Data Validation	NSL-KDD
^13^	DNN	Y	Single Data Validation	NSL-KDD
^14^	CNN	Y	Non IoT Validation	UNSW-NB15
^15^	RNN	Y	Single Data Validation	Bot-IoT
^16^	RF	N	Binary detection only	IoTID20
This paper	Ensemble	Y	Bot-IoT,IoTID20, CICIDS2018, NSL-KDD	N

## Proposed model

**Figure 2:**
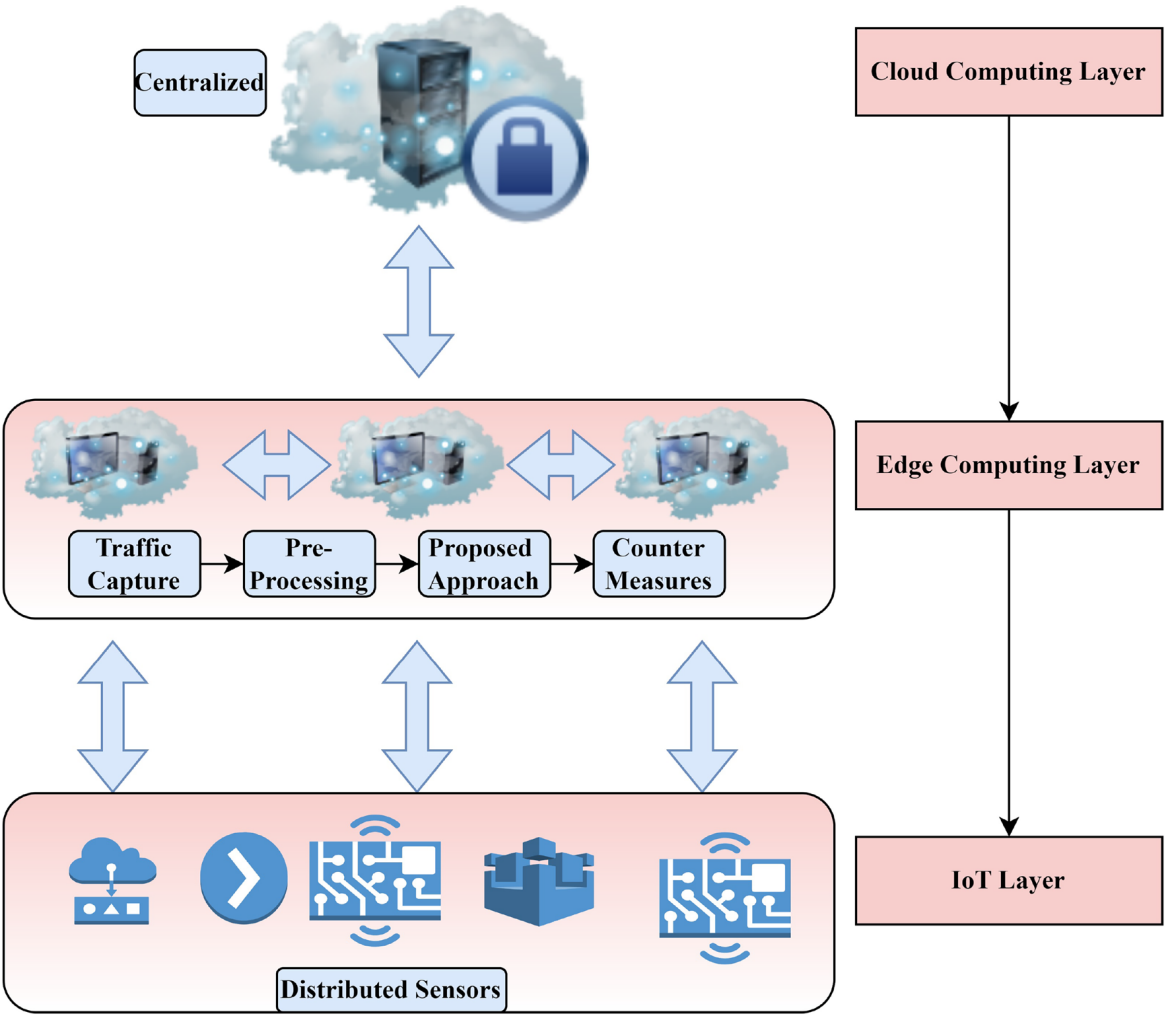
Proposed framework overview.

The capability to determine the specific category of an attack is vital procedure for countermeasures and decision-making, especially in light of the rising IoT and edge computational platform. Existing methods of multi-category detection are less reliable than the binary technique and have trouble recognizing certain kinds of attacks^22^. Conspicuously, the current study suggests a two-stage ensemble technique to detect and categorize the classes of attacks in IoT networks. Specifically, an edge-based IoT infrastructure is considered. To cause damage to innocent users, malicious actors try to break into or compromise infrastructural equipment. The suggested architecture is shown in Fig. 2 with the detection method deployed to the edge computing layer. The edge network nodes perform processing and storage closer to the physical system. As a result, potential dangers may be identified more quickly. In the best-case scenario, the edge node makes use of additional techniques to capture and pre-process network traffic. In addition, it may provide the information that the suggested method needs to determine whether and what kind of attack is being launched against the network. In this manner, the countermeasure system is alerted to the sort of attack that has been discovered and may take appropriate measures to thwart it. It is important to mention that edge computing environment is constraint by resources, specifically when new IoT devices are registered in the system. The current paper incorporates a hash value of the new IoT device or data transmitted. In other words, whenever new IoT node is registered in the system or whenever new data is to be transmitted, the edge node performs the conventional hashing technique to store the information locally. Using hashing technique, limited resources will be utilized at the edge node.

### Two-stage detection approach

**Figure 3:**
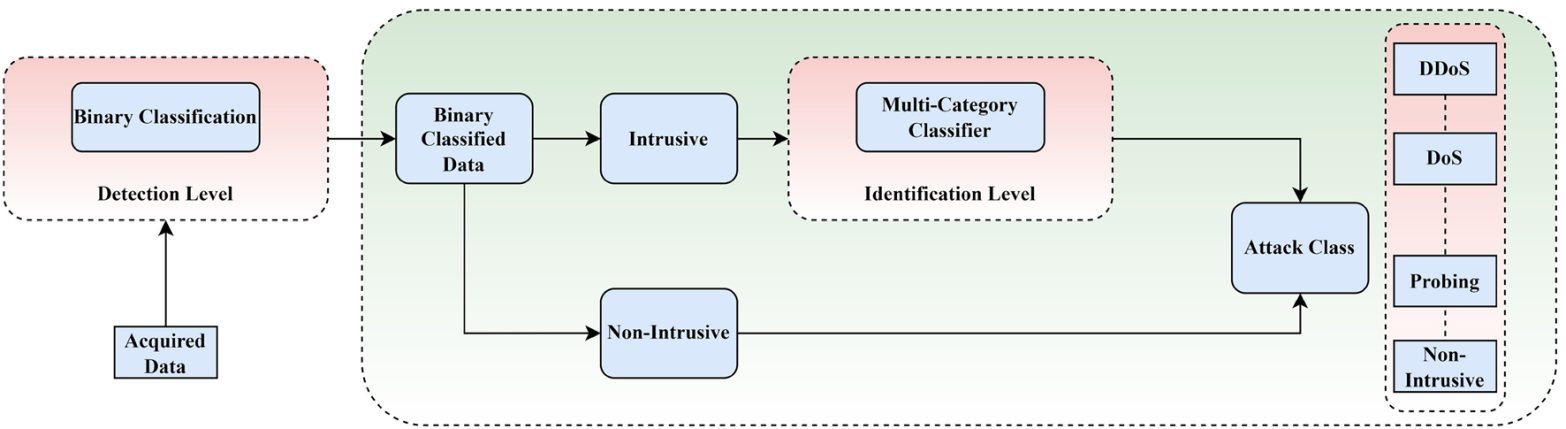
Proposed approach.

This subsection elaborates on the suggested method for detecting and identifying intrusions. The presented approach is shown in Fig. 3. Moreover, Algorithm 1 presents the pseudo-code for the proposed approach. Specifically, it consists of two stages of *detecting and identifying*. A binary classifier is used in the identification stage of traffic analysis. As a result, the IoT node will be labeled as “intrusive” or “non-intrusive” when all traffic flows it records. The gates automatically open for traffic that has been deemed safe. The second stage of the method, termed *identifying*, is applied to the invasive traffic. Here, a multi-category classifier reexamines the data to determine the broad category of incursion. False positives at the first level may be rectified at the second stage. Compared to analyzing the first step, the identification classification is an effective technique which needs processing power. Only if the first layer deems the incident disruptive will this strategy be put into action. Hence, this method enables the vast majority of valid network traffic to quickly and easily pass via binary determination. If the first stage identifies an invasive flow, it does not need to be dealt with immediately since a more thorough and delay analysis may be performed to define the attack type. It is crucial to correctly identify the intrusion at this point so that the countermeasure systems can respond appropriately to the various levels of threat.

**Figure:**
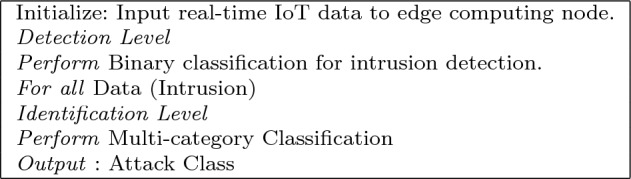
Algorithm 1: Intrusion Detection Procedure.

#### Detecting

Intrusion detection is the initial stage of the suggested technique. The collected traffic is then classified as either “invasive” or “non-intrusive” using an E-Tree classifier’s binary detection. Classifiers based on E-Trees^23^ are useful tools for many classification problems. An ensemble approach, E-Tree uses a large number of independently trained Decision Trees, resulting in a “forest”, to get final classification results. When it comes to data mining, DT is the best classification algorithm. Forecasting trees are a kind of prediction model described by Loef et al.^24^ as a recursive partitioning in subspaces. DTs are made up of nodes and take on a tree-like form. The root node represents the first partitioning decision that determines how the data will be divided. The data is partitioned further and farther down the tree by the intermediate nodes. The structure’s leaf nodes, therefore, stand in for the ultimate divisions. E-Trees’ primary tactic is to mix up which characteristics are utilized and where the cutoffs are set for those attributes to split the tree’s nodes. Hence, at each intermediary node, K resources are chosen at random from the whole collection of resources. Each decision tree must use some kind of mathematical criterion, such as the Gini index, to determine which of many possible resources should be used to partition the data. In the most extreme scenario, it constructs trees whose topologies are completely random and have nothing to do with the output values of the learning sample. For classification issues, the aggregated prediction is determined by a majority vote, whereas for regression problems, the aggregated forecast is determined by arithmetic means. The divisions of ET are computed in a completely random manner. A random subset of candidate qualities is employed, much as in Random Forest. With ET, on the other hand, cut points for each candidate characteristic are chosen at random rather than being optimized for discrimination. The dividing line is determined by randomly selecting the best of the produced cut points. The reasoning behind the technique is that the randomization of the cutoff and attributes, together with the ensemble mean, should result in a stronger variance reduction than would be achieved with a weaker randomization scheme. In the current work, several variables were considered. The Gini Index is employed as a criterion, there are a total of 11 DT estimators, a split sample size of 3 is required, and the number of attributes to be evaluated for improved splitting is equal to the square root of the number of existing attributes. As an intriguing alternative to traditional approaches, the ET classifier was selected to function in the initial detection stage because of its ability to achieve high performance in classification tasks with quick analysis.

#### Identification

**Figure 4:**
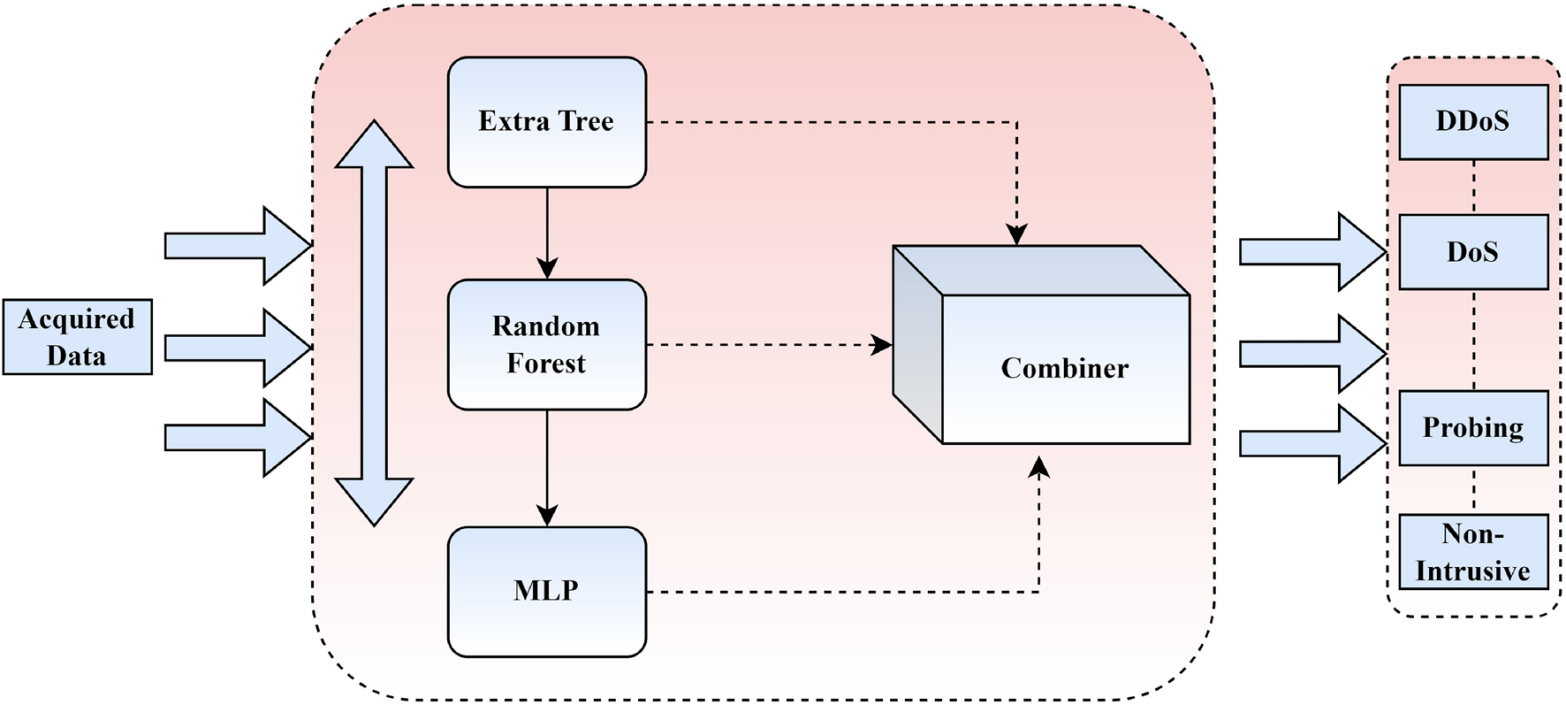
Ensemble classifier.

The second stage of the proposed method is identification, the goal of which is to evaluate traffic that has already been labeled as an intrusion and determine its category. At this stage, a detailed analysis is performed. It utilizes 3 different multi-category identification methods as part of an ensemble strategy. Because the first stage has already identified and released the regular events, there is no pressing need to react quickly to the events supplied for this second classification step, most of which are likely to be unwanted intrusions. Ensemble approaches aim to increase generalization and robustness over single classifiers by combining the classifications of conceptually diverse underlying machine learning classifiers^25^. Both of these approaches to determining the kind of attack utilize the same ET classifier, which was previously used in the first stage for binary classification. As shown in Fig. 4, this technique forms part of an ensemble approach with a DNN, Multilayer Perceptron, and RF. Two hidden layers with 148 neurons are employed in the MLP design. The activation function ReLU is used by the neurons in the network’s hidden layer, which performs the computations. If the input is larger than zero, the activation function will return that sum. If any of the inputs are negative, the result will be zero. As it is simpler to train and often produces excellent results, this simple function has been popular in many neural networks. One last component of this multi-category method is the RF algorithm. Ensemble techniques (E-Trees) using a collection of decision trees are similar to RFs. Using bagging techniques, several models are trained independently by utilizing different samples of data. Bagging and the CART algorithm form the basis of RFs. In contrast to bagging, RFs introduce a degree of randomization. In RFs, each tree is constructed using a distinct bootstrap sample of the data, and at each node, the best predictors for that node are used to divide the node. The purpose of the randomness sources is to reduce the forest estimator’s variance. The RF used in the method is 11 DTs in length and was developed with ET. Instead of having each classifier cast a vote for a single class, as the original RF method did, the system employed here combines classifiers by averaging their probabilistic prediction. A combiner based on the total maximum probability of the predictions is also implemented in the full technique shown in Fig. 4.

### Procedure: train

**Figure 5:**
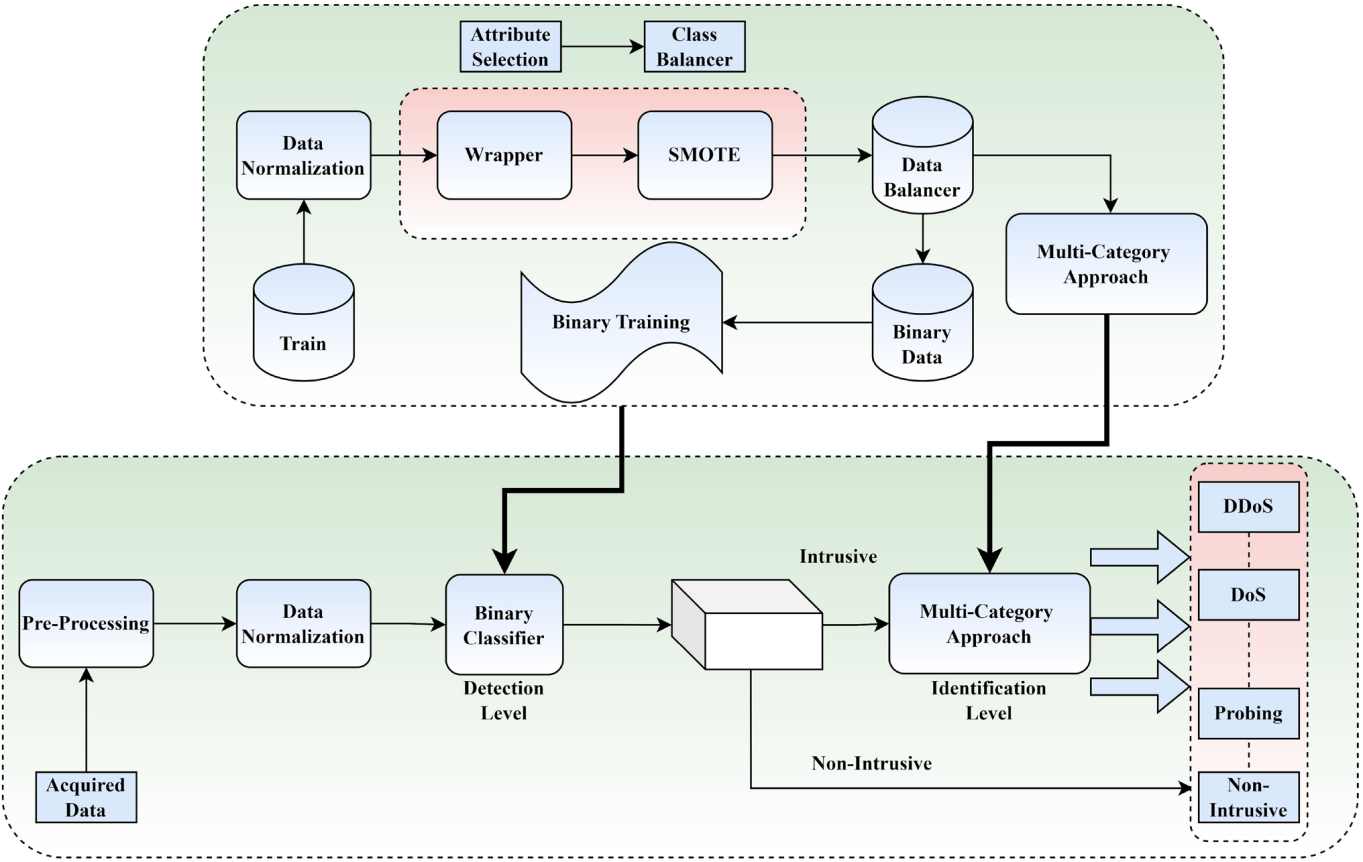
Training process approach.

To train the classification models, the method necessitates a dataset. Figure 5 shows the sequence of operations that must be carried out before the technique may carry out the detections. Before being used for training, the data is cleaned of invalid records, normalized, and formatted. Certain classifiers based on machine learning benefit from dataset standardization since it ensures that the resources they use are regularly distributed. The data were normalized using the conventional scaling approach as shown ahead.

$$\begin{aligned} a = \frac{y-v}{t} \end{aligned}$$

where y is the sample size, v is the mean, and t is the standard deviation. Using the statistic for each attribute in the dataset, we can calculate the mean and standard deviation. The method then applies an attribute selection strategy to narrow down the possible attributes of traffic to those that are most useful. Selecting the right collection of attributes for a classifier is the goal of attribute selection. To determine which attributes were most important, it used the recursive attribute elimination technique. The goal is to narrow down a list of potential candidates to a manageable size. The relevance of each resource is determined by training the classifier on the original set of resources. The resource with lower priority is thus dropped from consideration. To pick the optimal amount of attributes, this process is iterated recursively on the reduced collection. In this example, we aimed to choose the top 18 characteristics. The technique includes a balancing phase to make sure the models have enough data to comprehend the patterns of attacks that have a low incidence, which might create issues during training due to the difference in the percentage of traffic between the kinds of attacks. When dealing with imbalanced E-Trees, one approach is to generate additional instances for underrepresented groups. It is possible to create new data instances based on preexisting ones. The SMOTE technique was adopted since it is consistent with this strategy. For SMOTE to operate, a group of samples must be selected in resource space, a line must be drawn between the samples, and a new instance must be created at a location on the line. When SMOTE is applied to the original training data, a new dataset with evenly distributed classes is produced. The first and second-stage classifiers are then trained using the symmetrical dataset. Nevertheless, in the case of the first-step technique, binary-classified data is required due to the nature of the approach. The dataset is converted to binary before being utilized to train the first classifier in this fashion. At last, classifiers are trained for the two phases, yielding traffic classification models. After the model has been trained, it may begin processing fresh data from the network for analysis and classification. In practice, data is taken, pre-processed to extract just desired attributes, normalized, and fed into the trained technique, which then detects and identifies the kind of traffic.

## Experimental simulation

The primary criteria used to evaluate the effectiveness of the suggested strategy are outlined in this section. Furthermore shown and discussed are the outcomes of the experiments conducted. The viability of detection techniques in a real-world setting can only be determined via rigorous testing and analysis. Specifically, Intel i7 processor with 16GB RAM and 2.31GHz CPU cycle was used for deployment purposes. Moreover, Raspberry Pi v3 with 4GB RAM and 1.61GHz cycle was incorporated as the edge node for classification. The objective is to calculate an approximation of the methods’ actual accuracy. The following phrases will be used to classify the database’s event categories based on the tests performed. There are two types of detection errors: false positives (FP) and false negatives (FN). When an intrusion detection technology incorrectly identifies a negative event as positive, this is known as a false positive (FP). Negative occurrences that were accurately identified by the detection technique are called “True Negatives” (TN). Events that are identified as positive by an intrusion detection method are called True Positives (TP). It is feasible to evaluate the detection techniques developed by machine learning algorithms using a variety of metrics that may be calculated using the aforementioned terminology.

1. One such indicator is accuracy, which measures how many cases were properly categorized relative to the total number of occurrences. In extreme circumstances of social stratification, this may not be a helpful consideration. The accuracy is determined as Accuracy = $\frac{TN+TP}{TN+TP +FN+FP}$.
2. One other statistic often used to rank ML strategies is precision. This rate is the percentage of occurrences properly identified as positive relative to the total number of positive examples. Precision= $\frac{TP}{TP+FP}$.
3. Recall (REC): sometimes called sensitivity, it is the fraction of positive examples that were accurately labeled as such using Recall= $\frac{TP}{TP+FN}$.
4. The F1 measure is the average of the accuracy and memory scores, with the greatest possible score being 1 (for perfect accuracy and recall) and the poorest possible score being 0.
  $$\begin{aligned} F1-measure= 2*\frac{Precision *Recall}{Precision + Recall} \end{aligned}$$
5. An intriguing statistic for evaluating detection effectiveness on imbalanced data E-Trees is normalized accuracy. It is the cumulative sum of all of the classes’ recall rates.

### Using Bot-IoT for an evaluation

At the Cyber Range Lab at the University of New South Wales Canberra Cyber Center, a realistic network environment was designed and used to generate the BoT-IoT dataset. DoS, Recon, DDoS, and Steal Attacks are part of the ecosystem alongside regular traffic. 52 properties in the database describe various aspects of traffic. This data is exceptionally up-to-date and representative of actual IoT traffic at this time. To test the viability and effectiveness of the suggested method, several machine learning algorithms were assessed in experimental simulation. DNN, KNN, RF, and ET were among the traditional methods employed for this comparison. Table 2 demonstrates the wide range of results achieved by the DNN method. DoS attacks could be spotted, although the accuracy was poor overall. Using RF, KNN, or ET yielded intriguing and comparable findings. Although NB’s recall was good for the vast majority of attack types, its accuracy was only approximately 52% for Reconnaissance attacks, suggesting a large percentage of false positives. High detection and low false positives were shown by the suggested method, with recall and accuracy at almost 98%. The general detection results for each class in the database are summarized in Table 3. DNN performed worse. The outcomes from KNN, ET, RF, and NB were all comparable. Their average accuracy was thrown off because of their inability to identify theft attacks. A high detection rate across all kinds of threats allowed the suggested technique to attain extraordinarily high balanced average detection. With a mean prediction time of 15.12 s, the method was the third fastest, behind only DNN and KNN. The algorithms’ processing times for classifying dataset events are included in the prediction time. The suggested method applies an ET at the first level of detection, allowing for considerably quicker identification of benign traffic by analyzing simply the traffic at the first level. So, it is assumed that the suggested method’s forecast time for just positive occurrences would be similar to that achieved by the ET method. On the other hand, a more robust strategy that results in a longer forecast time identifies invasive events at the first level and submits them for study at the second level. Yet, because they have already been recognized as invasive, there is no pressing need to categorize this communication, since it may be stopped without consequence. As a result of the adjustable reaction time at the second level, traffic flagged as malicious at the first level may be sent to the cloud, where a more complicated analysis can be done, relieving pressure on edge resources. Table 4 compares the experimental results achieved by the suggested technique with the results acquired by state-of-the-art approaches using the Bot-IoT dataset. Additionally, Telikani et al.^26^’s work ensured that detection rates for all attacks remained over 97%. The method provided in^27^ was effective across all three groups but did not offer a benign traffic detection rate since it lumped DDoS and DoS into a single category. Good performance was attained by Achiluzzi et al.^28^ and Ramesh et al.^29^, however they struggled to identify Data Theft. Nevertheless, the method presented by Hasan et al.^30^ was successful in identifying attacks but struggled to identify innocuous traffic, suggesting an increase in false positives. The suggested method outperformed all others in detecting various types of attacks.

**Table 2:** Comparative analysis Bot-IoT data set (all values in %).

Attack	KNN	KNN	RF	RF	DNN	DNN	ET	ET	NB	NB	Proposed	Proposed
	REC	PRE	REC	PRE	REC	PRE	REC	PRE	REC	PRE	REC	PRE
DDoS	98.25	99.36	99.25	95.26	96.15	95.32	99.26	94.25	96.25	96.96	99.95	99.94
Recon	95.65	94.25	96.36	94.15	96.25	94.54	96.66	95.55	94.66	95.15	98.9	98.9
DoS	96.36	94.15	94.02	93.21	96.25	96.14	96.45	96.66	94.58	94.25	99.32	99.15
Theft	96.26	96.66	94.25	94.36	95.15	91.25	94.25	96.14	95.36	94.14	98.65	98.36
Benign	98.25	98.15	98.14	98.65	98.25	94.26	98.25	98.25	98.25	98.15	99.36	99.25

**Table 3:** Overall comparative analysis BoT-IoT data.

Method	Accuracy	Balanced accuracy	PRE	Delay
DNN	98.95	96.56	94.56	20.15
RF	98.95	98.45	98.15	29.2
ET	98.25	98.25	98.65	65.23
KNN	98.65	96.26	94.15	0.99
NB	98.14	96.26	98.14	9.26
Proposed	99.95	99.59	99.14	1.23

**Table 4:** Comparative analysis with BoT-IoT data.

References	DDoS	Benign	DoS	Theft	Recon.
^14^	93.26	94.26	95.26	94.15	96.26
^13^	94.12	94.26	94.26	95.15	93.25
^12^	91.25	96.36	95.26	94.15	93.25
^11^	92.36	91.23	91.25	93.33	92.2
This Work	97.23	96.23	97.32	97.12	97.12

### IoTID20 assessment

The IoTID20^31^ dataset contains information on the network activity of IoT devices and the kinds of linked systems seen in Smart Homes. CCTV cameras are one type of gadget in the monitored infrastructure. A wide range of attacks, including DoS, Botnet Mirai, Man-in-the-Middle, and probing, have been documented. Table 5 displays the results achieved using the suggested method as well as the other machine-learning techniques. The NB method performed the poorest because, despite its near-perfect detection rate for DoS attacks, it was unable to accurately distinguish between malicious and benign traffic, leading to a significant number of FP. Identified regular traffic and Mirai-based, and scanning attacks at rates exceeding 92% with the other approaches. The percentage of MITM-related traffic discovered by DNN was more than 85%, by KNN 92%, by RF 92%, and by ET 94%. In this situation, the detection was improved by more than 98% according to the suggested method. As a result, the suggested method consistently achieved a hit rate of 98% or greater, even for benign traffic, proving its high accuracy and low false-positive rate. The general detection results for each class in the database are summarised in Table 6. As compared to the balanced average general accuracy, the suggested method emerges as a clear victor. It hit 99% due to the method’s uniformly excellent results across all subject areas. The normalized accuracy (balanced) value for the RF, KNN, and ET techniques, on the other hand, remained between 94% and 97% because of the low detection rate of MITM attacks. The KNN’s negative effect on the prediction time is shown by the fact that it took more than 500 s to classify the events in the data set. The time required for the suggested method was determined to be more than 3.2 s. Time is reduced when dealing with benign traffic categorization since just the first level of analysis is required for this kind of traffic. Given that the vast majority of traffic in the actual world is completely benign, the method is capable of producing predictions with accuracy near ET.

**Table 5:** Comparative analysis IoTID20 data set (all values in %).

	KNN	KNN	RF	RF	DNN	DNN	ET	ET	NB	NB	Proposed	Proposed
	REC	PRE	REC	PRE	REC	PRE	REC	PRE	REC	PRE	REC	PRE
Mirari	97.25	97.36	97.75	97.76	96.17	96.62	96.66	94.25	97.25	97.76	97.95	97.97
Benign	95.68	94.56	97.76	98.15	98.25	96.56	96.76	97.57	97.86	95.56	98.9	98.9
DoS	96.36	94.15	96.62	93.66	96.65	96.16	96.45	97.66	96.58	94.65	96.32	96.15
Scan	96.66	96.56	95.25	94.55	95.55	95.25	94.55	96.16	96.36	96.64	98.66	98.66
MITM	97.25	98.17	98.17	97.65	97.75	97.26	98.75	98.25	97.25	98.75	99.36	99.25

**Table 6:** Overall comparative analysis IoTID20 data.

Method	Accuracy	Balanced Accuracy	PRE	Delay (s)
DNN	98.75	97.56	97.56	25.15
RF	98.85	97.45	97.15	265.25
ET	97.25	98.75	96.65	64.23
KNN	97.65	97.26	97.15	3.49
NB	96.17	97.26	97.14	66.56
Proposed	99.00	99.50	99.04	1.53

### Comparison using NSL-KDD

Several recent publications^32^ evaluate intrusion detection techniques using the NSL-KDD1 database. There are 42 properties, or resources, associated with each instance of a base class, 31 of which are the observable attributes of network connections gleaned via packet analysis. The instance label specifies whether the occurrence is an attack or a typical occurrence. The total number of records in the NSL-KDD dataset is more than 66,215, as shown in Table 7. Results for detecting and identifying attacks using the NSL-KDD database are shown in Table 8 for both the suggested strategy and traditional machine learning approaches. The results of these studies show that the NB approach performed poorly, with low accuracy across all attack classes. In terms of detecting DDoS and probing attacks, the DNN method performed well. However, no accuracy is registered for U2R attacks. Identifying both innocuous traffic and DoS and probing attacks was easy using the RF, KNN, and ET methods. Also, the four methods detected R2L attacks with a mean of 98% success rate. U2R attacks were more difficult to detect. 90% detection was achieved using the NB method, but with an accuracy of less than 2%. Around 82.2% of U2R attacks were spotted by the ET, while 80% were seen by the RF, DNN, and KNN methods. The suggested method even managed to boost the detection rate to 88%. In Table 9, we can see how different classes do on average when it comes to general detection. Only NB failed to achieve the minimum level of mean accuracy required of all other methods. It is noteworthy to evaluate the BACC (normalized accuracy), a measure that takes into account the disparity across classes, in addition to the average accuracy. The suggested method was shown to be superior to all the others tested. This statistic matters because it provides a more accurate picture of the typical rate of attack detection. Although DNN, KNN, and RF all managed to go below 88% accurate on average, the suggested method managed to get close to 96%. The ET method was somewhat less effective than the suggested strategy, with a balanced mean accuracy of about 94%. The suggested method achieved an impressive prediction time of 3.61 s when applied to the dataset, placing it near the RF, DNN, ET, and NB methods. This is mostly because there is more benign traffic than in earlier dataset. Results from the experiment using the NSL-KDD database were compared with those from state-of-the-art methods, including the one provided in this paper. The outcomes of these methods in each attack category are shown in Table 10. Recall rates are provided for the works, showing how much of each category was identified. As can be shown, the suggested method successfully detected over 98% of both benign traffic and malicious DoS and Probing attempts. For R2L attacks, the suggested method achieved about 97% detection, ranking it in second place behind the method of Hassan et al.^33^, which achieved approximately 98%. Unfortunately, U2R attacks have eluded detection in this investigation. There are not many flows associated with U2R attacks. Both cutting-edge and traditional machine learning systems struggle to spot these attacks. Around 88.5% detection was achieved using the suggested method, second only to the method developed by Syed et al.^34^, which achieved 87.5% detection. Nevertheless, this method is incapable of detecting typical flows. In this case, a low rate suggests that the method has a high rate of false positives. This is a significant issue with current methods of intrusion detection. Around 98.9% of normal flows may be detected using the suggested method. As a result, it can identify attacks while producing negligible amounts of FP.

**Table 7:** NSL-KDD data attack class.

Class	Train	Test
DoS	66,215	41,255
Benign	46,521	21,541
R2L	1021	295
Probing	12,365	1025
U2R	65	35

**Table 8:** Comparative analysis NSL-KDD data set (all values in %).

	KNN	KNN	RF	RF	DNN	DNN	ET	ET	NB	NB	Proposed	Proposed
	REC	PRE	REC	PRE	REC	PRE	REC	PRE	REC	PRE	REC	PRE
DoS	92.35	92.36	92.35	93.26	92.22	92.62	93.63	93.25	93.35	93.46	94.95	94.44
Benign	95.68	95.56	95.56	95.15	95.25	94.56	94.46	94.54	97.86	94.56	94.44	94.9
R2L	93.36	94.35	93.62	94.46	94.65	94.16	94.45	94.46	96.58	93.65	93.32	96.15
Probing	93.66	93.56	93.25	94.35	95.53	93.35	93.55	93.13	93.33	93.44	94.46	94.66
U2R	96.65	96.67	96.17	95.65	97.55	95.56	95.75	94.25	96.25	96.75	96.36	96.25

**Table 9:** Overall comparative analysis NSL-KDD data.

Method	Accuracy	Balanced accuracy	PRE	Delay (s)
DNN	92.35	93.56	94.46	0.36
RF	92.83	92.44	94.15	5.15
ET	92.25	92.75	93.35	4.23
KNN	95.65	94.46	94.35	1.19
NB	95.57	95.26	97.14	0.56
Proposed	98.70	98.88	98.04	1.13

**Table 10:** Comparative analysis with NSL-KDD data.

References	DDoS	Benign	DoS	R2L	U2R
^14^	95.24	93.26	93.26	93.44	–
^13^	93.12	93.26	93.26	93.13	94.25
^12^	94.25	94.36	–	93.15	–
^11^	93.36	93.23	93.25	–	93.2
This Work	98.83	97.83	98.32	98.16	98.12

### Assessment using CICIDS2018

**Figure 6:**
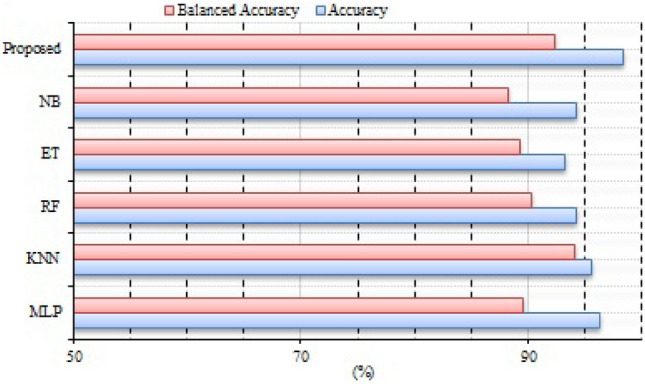
Mean analysis with CICIDS2018 data.

**Figure 7:**
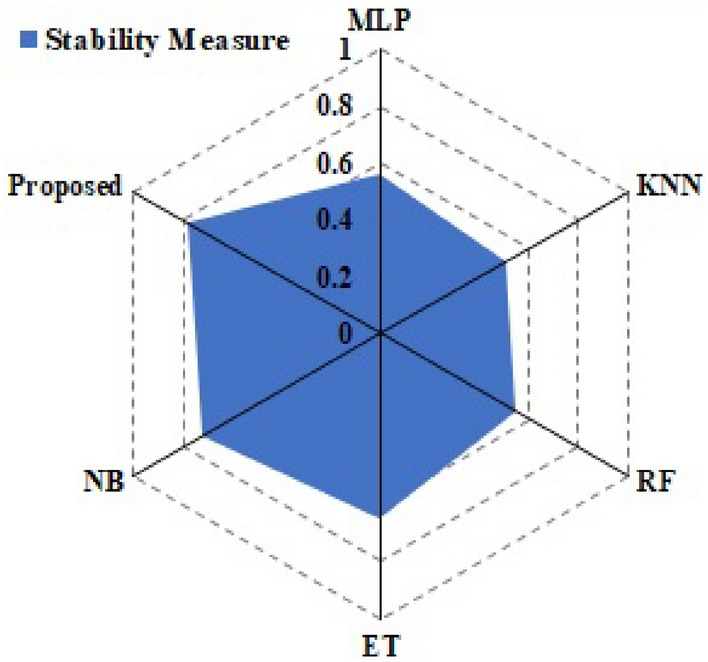
Stability comparison.

The CICIDS2018 dataset was used to assess the suggested method. The goal of the CICIDS2018 project is to provide a method for producing a variety of educational data from user profiles that include a variety of events and network-observed behaviors. Brute force, DDoS, DoS, Web, and infiltration are all included in this package. The sheer volume of information makes it impractical to analyze the whole database, thus a sample size of 8% is often used instead. The number of records within each class was maintained throughout the random extraction of 8%. Results for both the suggested method and traditional machine learning techniques for using the CICIDS2018 database to detect and identify attacks are shown in Table 11. Taking into account the recall measure, it is clear that the NB approach fared poorly in this experiment due to the large differences in performance across attacks. This indicator shows how many instances are in a certain class as a percentage of all instances. As a result, it shows the relative amounts of various types of attacks that were uncovered. Recall rates varied greatly throughout NB technique types of attacks. DDoS, DoS, and Botnet attacks were detected successfully with the help of the suggested method using the DNN, ET, KNN, and RF approaches. Also, they were able to successfully identify over 90% of brute force attacks. Web attacks, infiltration attacks, and other methods were made more difficult to detect. In all types of attacks, the suggested method has detection rates on par with or greater than previous machine-learning methods. The overall findings in Table 12 reflect the registered results. When comparing the balanced accuracy rates, the suggested method is better. This measure accounts for the disparity in attack types. Rates of about 96% were shown using the RF, KNN, and ET methods. The suggested method was successful in overcoming them, with a 96% balanced average accuracy being achieved. The suggested method achieved the fastest classification time on the dataset. This is mostly because there is more benign traffic than in earlier data E-Trees. When looking at the average ACC and BACC rates in Fig. 6, the suggested method stands out as the best option. The method’s ACC was high, ranking among the best and on par with that of DNN, RF, KNN, and ET. It is stressed that NB’s performance was subpar. The suggested method achieved the greatest percentage of accuracy when compared to the average of all other methods. Again, the KNN, and DT methods all achieved the same high level of accuracy. The suggested method was tested experimentally and compared to both state-of-the-art and machine-learning methods. The viability and robustness of the suggested technique for intrusion detection were proved across a wide range of situations in an assessment conducted using the IoTID20, Bot-IoT, NSL-KDD, and CICIDS2018 data. The suggested method was effective as compared to state-of-the-art methods on all databases. In addition, the stability analysis shown in Fig. 7 demonstrates that the presented technique is more durable as compared to state-of-the-art techniques.

**Table 11:** Comparative analysis CICIDS2018 data set (all values in %).

	KNN	KNN	RF	RF	DNN	DNN	ET	ET	NB	NB	Proposed	Proposed
	REC	PRE	REC	PRE	REC	PRE	REC	PRE	REC	PRE	REC	PRE
DoS	93.61	92.66	92.61	96.26	92.22	92.62	96.66	96.21	96.61	92.36	93.91	93.33
Benign	96.68	91.16	91.16	91.11	91.21	93.16	94.46	94.14	97.86	94.16	94.44	94.9
Botnet	96.66	94.61	96.62	94.46	94.61	94.16	94.41	94.46	96.18	96.61	96.62	96.11
Web Attack	96.66	96.16	96.21	94.61	91.16	96.61	96.11	96.16	96.66	96.44	94.46	94.66
Infiltration	96.61	96.67	96.17	91.61	97.11	91.16	91.71	94.21	96.21	96.71	96.66	96.25

**Table 12:** Overall comparative analysis CICIDS2018 data.

Method	Accuracy	Balanced Accuracy	PRE	Delay
DNN	95.55	95.54	93.53	24.15
RF	95.95	88.65	94.13	44.2
ET	96.75	98.25	98.65	55.23
KNN	94.65	96.26	94.15	45.99
NB	93.14	94.56	95.14	94.26
Proposed	97.97	97.79	98.14	3.23

### Time complexity

We investigate the time required to categorize an occurrence. The first and second stages, binary detection and ensemble method, respectively, need the most computer resources. Costs associated with other comparisons, assignments, and constant totaling are ignored. The suggested method utilizes an E-Tree for preliminary analysis. The expense of making a prediction is proportional to the number of comparisons conducted at each node in the tree. Each level only has one comparison done at the level node level. The proposed E-Tree consists of 8 trees, each of which has a depth q that is set during training. The ET complexity is given as ET (complexity) = (8$*$q). RF, ET, and MLP are employed together as an ensemble approach at the advanced stage. The ET used in the ensemble approach is structurally identical to the ET employed in the first level, and the RF method is very similar to ET other from some minor randomization variations. Each of the 8 trees in the planned RF has a depth of q. This means that the prediction complexity of ET and RF will be the same. A total of g neurons are spread over two hidden layers in the proposed DNN architecture, whereas n neurons are used in the output layer. The number of neurons in the input layer, e, is equal to the number of attributes or dimensions that must be used to categorize the incoming event. Each node in one layer has an associated weight with all nodes in the following layer since the DNN is completely linked. The inputs to each hidden neuron are linearly combined, and then an activation function is applied. Consequently, e$*$g operations are executed in the first concealed layer. Operations of the kind g$*$g are carried out in the second hidden layer. Also, the neurons in the output layer linearly combine their inputs and then apply an activation function. Our design can only do g$*$n operations since there are only two neurons in the output layer. Hence, we may model computational complexity as Complexity (Level 1) = (e$*$g+g$*$g+g$*$n). Thus, the second-level difficulty is computed as Complexity (Level 2) = Complexity (Level 1) + Complexity (ET) + Complexity (RF). ET, and DNN all contribute to the overall complexity. There is also a complicated mix of predictions that is outside the scope of this investigation. It follows that the ideal scenario for a traffic instance’s forecast time happens when the traffic is immediately categorized as benign at the first level. In this scenario, we skip over the second-level detection prediction. As a result, the sole complexity is computed. Complexity = O(q), where q is the depth of the trees, is a reasonable approximation for the best-case performance of this method. Different trees will have different starting depths, and they will get deeper. Deep E-Trees may result in massive tree structures. As a result, experimenting with depth restrictions may be a fun idea for future projects. In the worst situation, the first-level ET labels some traffic as invasive, prompting further labeling using the second-level multi-category ensemble approach. Hence, the complexity is equal to the sum of the first-level complexity and the second-level complexity. Due to their relationship with the structure of the classifiers rather than the size of the entry, the number of dimensions (e) of an event to be categorized, the number of neurons in each hidden layer (g), and the number of neurons in the output layer (n) may be regarded constants. Hence, we may think about the whole complexity = O(q). The training stage will determine how deep the trees will be since it’s the sole variable that may lead to more operations. By setting maximum depth limitations using criteria, we may manage tree growth and save expenditures. In addition, the batch size may be thought of as the input size (o) when analyzing batch flow detection. By carefully examining all of these moving parts, we have concluded that this method can indeed help the issue scale.

### Statistical analysis

**Figure 8:**
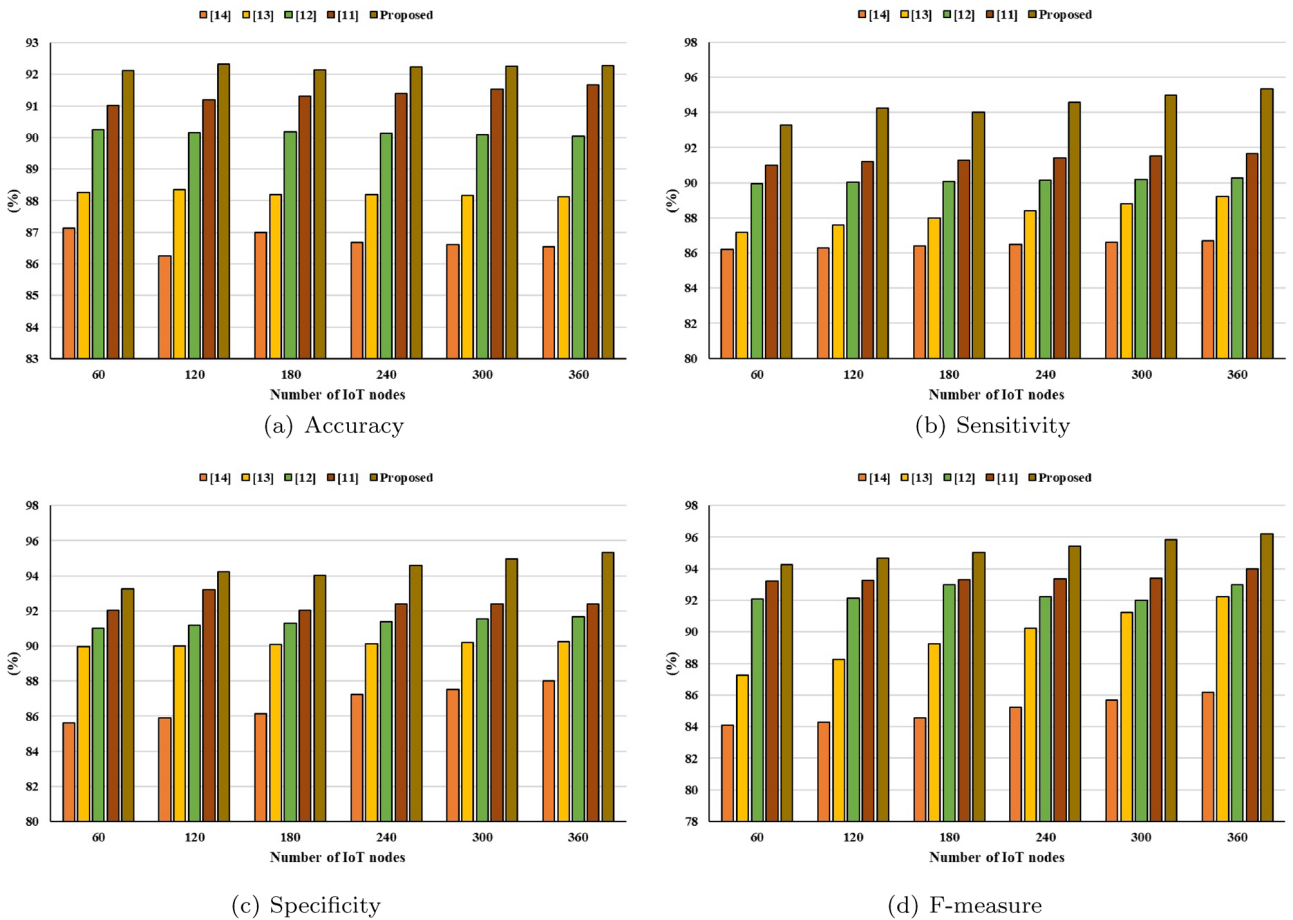
Statistical analysis.

**Figure 9:**
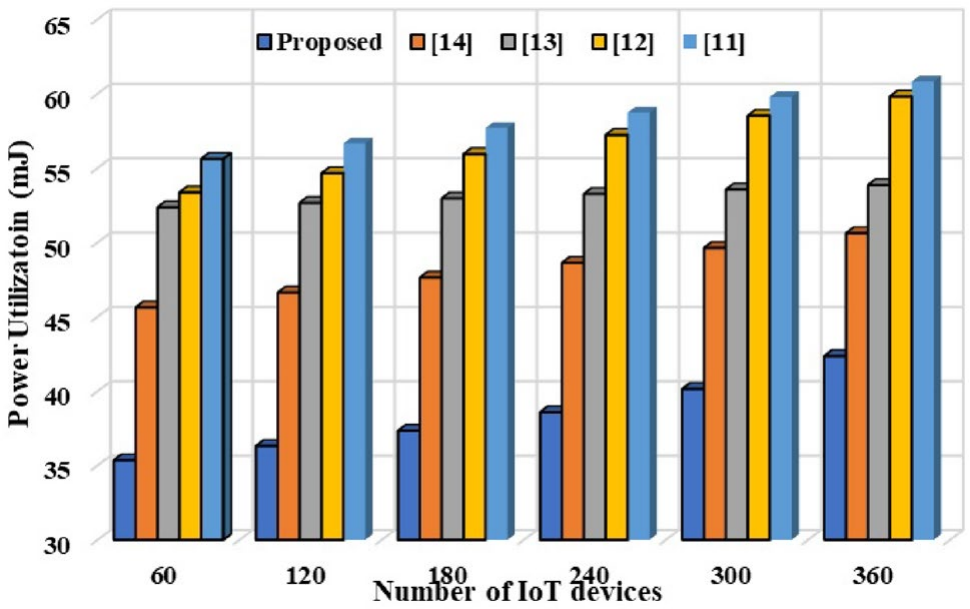
Energy consumption analysis.

This section discussed the statistical analysis of the proposed model in terms of scalability aspects and resource utilization. For validation of the scalability and generalization, the proposed model is deployed with different number of IoT nodes using IOTIFY simulator. IoT nodes have heterogeneous infrastructure for ensuring general view of the proposed model. The performance is estimated in terms of statistical parameters of Accuracy, Sensitivity, Specificity, and F-Measure. Results are depicted in Fig. 8. It can be seen that, in the current scenario, the proposed model is able to achieve high accuracy of 92.65% as compared to state-of-the-art techniques. Moreover, higher sensitivity (90.12%), and specificity (91.32%) further demonstrate the effectiveness of the proposed model. In addition to scalability, the proposed model is assessed for power utilization for different nodes. The detailed comparative results are depicted in Fig. 9. It cane be seen that in the current scenario, the proposed model incorporates minimal energy of 56.21mJ as compared to state-of-the-art techniques. Based on the aforementioned results, the proposed model is better and effective.

### Discussion

The proposed method can adapt to evolving security threats through several mechanisms.

1. The use of ensemble learning techniques, such as the combination of RF, E-Tree, and DNN, enables the intrusion detection system to adapt to new and emerging threat patterns by leveraging the strengths of multiple models. This allows the system to effectively identify and respond to previously unseen attack types.
2. The incorporation of edge computing in the intrusion detection architecture enhances adaptability by enabling real-time analysis and response at the network’s edge. This approach can facilitate rapid adaptation to evolving threats by minimizing latency and enabling timely threat detection and mitigation.
3. The use of dynamic feature selection methods and attribute detection mechanisms can enable the system to adjust its focus based on evolving threat landscapes, ensuring that it remains effective in identifying new intrusion patterns.

#### Potential challenges

1. One potential challenge in applying the proposed methodology in real-world scenarios with varied and dynamic conditions is the diverse nature of IoT environments, which can encompass a wide range of device types, communication protocols, and network topologies. To address this challenge, the methodology can be adapted to include a flexible and modular design that allows for customization to different IoT environments. This could involve the development of adaptable algorithms and models that can effectively operate across diverse IoT setups.
2. Another challenge is the dynamic nature of IoT traffic and network conditions, which can lead to fluctuations in network traffic patterns and varying levels of data volume. A potential solution to this challenge is the implementation of dynamic reconfiguration capabilities within the intrusion detection system. This would allow the system to adjust to changing network conditions and traffic patterns in real time, ensuring consistent performance and accuracy.

#### Practical implementations aspects

Practical implementation aspects that could enhance the proposed model’s applicability include considerations for real-world deployment of the proposed intrusion detection system in IoT environments. Specific aspects include;

1. *User-Friendly Interfaces*: Designing intuitive user interfaces and management tools that simplify the deployment, configuration, and monitoring of the intrusion detection system. This could include developing dashboards, APIs, or command-line interfaces that enable users to interact with and manage the system effectively.
2. *Interoperability and Standards*: Ensuring compatibility and interoperability with existing IoT platforms and communication protocols to facilitate seamless integration with diverse IoT ecosystems. Adhering to industry standards and protocols can enhance the system’s compatibility and ease of deployment in real-world IoT environments.
3. *Network Dynamics and Latency*: Considering the impact of network dynamics, potential communication latency, and intermittent connectivity within IoT networks. This involves designing the system to handle variations in network conditions and ensuring robustness in the face of communication delays or disruptions.

### Limitation and future work

As a limiting aspects, the suggested ensemble intrusion detection and identification technique was developed and tested to detect attacks in the IoT and cloud computing platforms. While the suggested method performs well, it is important to note that IoT is still susceptible to attacks such as wormholes, sinkholes, and forwarding attacks that are not addressed in the study. The countermeasures module is only demonstrated theoretically, which is another shortcoming. Dependence on specific datasets for validation might not represent all types of IoT traffic. However, the generation of synthetic datasets that mimic real-world IoT traffic patterns and attack behaviors can help supplement the validation process, providing a more comprehensive evaluation of the intrusion detection system’s performance across diverse scenarios. Furthermore, conducting field trials or real-world testing in different IoT deployment settings can offer valuable insights into the system’s effectiveness in detecting intrusions across varied environments. By combining these approaches, the research can mitigate the limitation of dataset dependence and enhance the system’s representation of diverse IoT traffic, ultimately improving its applicability and robustness in real-world IoT deployments. In addition to the current study, future research could explore the development of real-time response mechanisms to mitigate IoT attacks, such as automated incident response systems and adaptive security measures. Furthermore, future work could focus on methods for detecting and responding to dynamic adversarial attacks targeting IoT devices, including the development of adaptive intrusion detection techniques. Lastly, there is a potential for research into techniques for privacy-preserving intrusion detection in IoT environments, with a focus on maintaining data privacy while effectively identifying and responding to intrusions.

## Conclusion

IoT-inspired edge computing is a vital platform that is susceptible to attacks. The IoTID20, Bot-IoT, NSL-KDD, and CICIDS2018 data were used to test the proposed intrusion detection and identification technique with two stages of analysis. The suggested method was tested experimentally and compared to both state-of-the-art and machine-learning methods. The suggested method was more effective than the state-of-the-art methods on all databases. When E-Tree is used for a binary analysis in the first stage of detection, benign traffic may be released quickly while events that cannot be classified as benign are subjected to a more thorough examination using an ensemble technique. The network administrator and defense systems benefit from more detailed information when the attack type is known. Future research efforts will be directed at creating countermeasure mechanisms that are compatible with differentiated courses of action for various types of intrusion.

## Data Availability

The data used to support the findings of this study are available from the corresponding author upon request.
